# Immune-driven biotransformation of gold nanosheets by human primary neutrophils

**DOI:** 10.1039/d6na00592f

**Published:** 2026-08-28

**Authors:** Soumyadeep Poddar, Vishal Nayak, Deeksha Suvarna, Pavithra Kurungottu, Sandeep Shrivastava, Rajendra Kurapati, Srinivasa Reddy Bonam

**Affiliations:** a Vaccine Immunology Laboratory, Department of Applied Biology, CSIR-Indian Institute of Chemical Technology Hyderabad 500007 India bonamsr.iict@csir.res.in rkurapati@iisertvm.ac.in; b Academy of Scientific and Innovative Research Ghaziabad 201002 India; c School of Chemistry, Indian Institute of Science Education and Research Thiruvananthapuram Maruthamala P.O., Vithura Thiruvananthapuram 695551 Kerala; d CSIR-Centre for Cellular and Molecular Biology Uppal Road Hyderabad 500 007 India

## Abstract

The presumed chemical inertness of gold nanomaterials underpins their biomedical application, yet their structural fate within the oxidative milieu of innate immune cells remains uncharacterised. Here, we show that sustained engagement of two-dimensional gold nanosheets (AuNS) with primary human neutrophils over 14 days induces progressive crystalline domain disruption and sulfur incorporation within degraded regions, resolved by transmission and scanning electron microscopy with coupled energy-dispersive X-ray analysis. Pharmacological dissection using inhibitors of myeloperoxidase (4-aminobenzoic acid hydrazide, 4-ABAH), NADPH oxidase NOX1/4 (Setanaxib; GKT137831) and 1-methylpropyl 2-imidazolyl disulfide; thioredoxin-1 (PX12) demonstrates that degradation requires convergence of parallel oxidative routes (MPO/HOCl and Fenton-like) with Trx-1-mediated thiol capture of Au^+^ as Au–S-Trx. PX12 abolishes the diagnostic Au–S signature without restoring gold content, whereas simultaneous blockade of all three axes returns gold to near-pristine levels with near-complete nanosheet persistence. Transcriptional profiling further reveals coupling between oxidative burst activity and inflammatory signalling. These findings redefine the biological stability of gold nanostructures under physiologically relevant inflammatory conditions.

## Abstract 2

Human neutrophils biodegrade gold nanosheets *via* convergent MPO, NADPH oxidase, and thioredoxin-1 pathways, forming Au–S adducts; blocking all three pathways preserves nanosheet structure and gold content.

## Graphical abstract

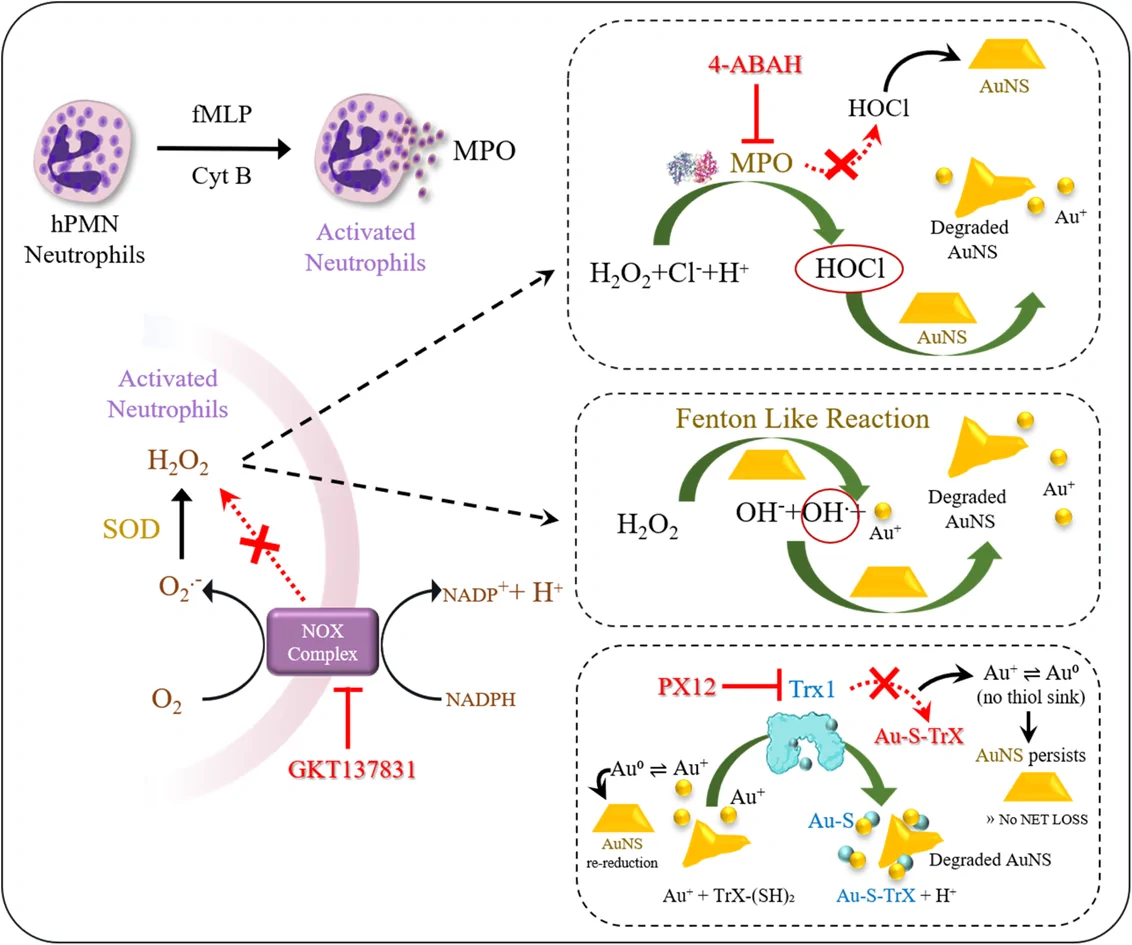

## Introduction

Gold nanomaterials have attracted substantial interest for biomedical applications, including drug delivery, imaging, and photothermal therapy, due to their unique optical and physicochemical properties.^1–3^ Despite their extensive exploration, the long-term fate of gold nanostructures in biological systems remains incompletely understood. Gold is traditionally regarded as chemically inert, and therefore, many studies assume that gold nanomaterials remain structurally stable in physiological environments. Recent studies have challenged this assumption by demonstrating that intracellular oxidative processes can lead to the biotransformation of gold nanoparticles.^4,5^ Long-term observations in fibroblasts revealed that gold nanoparticles can undergo gradual oxidative dissolution followed by recrystallisation into nanoscale assemblies termed aurosomes, which are often associated with sulfur species and lysosomal metabolism.^1,3,5,6^ While these findings provide insight into intracellular degradation mechanisms, the potential role of immune cells in mediating degradation of gold nanomaterials in the bloodstream or tissue immune environment remains largely unexplored. Neutrophils represent the first line of innate immune defence and are the first cells recruited to the site of injury/infection, equipped with potent oxidative enzymes, including myeloperoxidase (MPO), capable of generating reactive oxidants such as hypochlorous acid (HOCl).^7^ Beyond MPO, neutrophils also generate superoxide and hydrogen peroxide (H_2_O_2_) through NADPH oxidase (NOX) complexes, and regulate intracellular redox homeostasis *via* thioredoxin-dependent pathways.^8^ These reactive species have collectively been shown to degrade various nanomaterials, including carbon nanostructures and graphene derivatives.^9–11^

The multi-enzyme oxidative arsenal of neutrophils raises the possibility that 2D gold nanosheets (AuNS) degradation is not driven by a single oxidative pathway but rather by the convergent activity of MPO, NOX-derived reactive oxygen species, and thioredoxin-mediated redox processes. Investigating the relative contributions of these pathways through selective pharmacological inhibition is therefore essential for establishing the mechanistic basis of nanosheet degradation and for predicting nanomaterial fate under inflammatory conditions. Furthermore, given that nanomaterial-immune cell interactions can modulate cytokine secretion and inflammatory signalling, characterizing the immunomodulatory consequences of AuNS exposure and inhibitor treatment provides an important additional dimension for understanding the biological impact of gold nanomaterial degradation.

In this study, we investigated whether human neutrophils can mediate the degradation of AuNS.^12–14^ Using transmission electron microscopy (TEM) and elemental analysis, we examined the structural evolution of AuNS during repeated exposure to freshly isolated human neutrophils. The roles of MPO, NADPH oxidase, and thioredoxin-1 in the degradation process were assessed using a triple-axis pharmacological inhibition strategy employing 4-ABAH, GKT137831, and PX12, respectively. In addition, pro- and anti-inflammatory cytokine profiles were quantified to evaluate the immunomodulatory consequences of inhibitor treatment in this model.

## Methods

### Neutrophil isolation from human peripheral blood

Human peripheral blood was obtained from healthy donors and processed immediately after collection. Polymorphonuclear (PMNs) neutrophils were isolated using Ficoll–Paque density gradient centrifugation.^15^ Briefly, whole blood was carefully layered over Ficoll–Paque solution and centrifuged at 400×*g* for 30 minutes at room temperature without brake. Following centrifugation, the PMNs cell layer located beneath the mononuclear cell interface was collected. Residual erythrocytes were removed by hypotonic lysis using ammonium chloride buffer, and the cells were washed twice with phosphate-buffered saline (PBS). Isolated neutrophils were resuspended in RPMI-1640 culture medium supplemented with 10% fetal bovine serum (FBS) and maintained at 37 °C in a humidified incubator containing 5% CO_2_. Cell viability was assessed using trypan blue exclusion and was consistently above 95% prior to experimental use.

### Neutrophil activation

To induce myeloperoxidase (MPO) release, isolated PMN neutrophils were stimulated using *N*-formyl-methionyl-leucyl-phenylalanine (fMLP) in the presence of cytochalasin B (Cyt B). Initially incubated with Cyt B (5 µg mL^−1^) for 5–10 minutes at 37 °C to enhance degranulation. Subsequently, cells were stimulated with fMLP (100 nM) to trigger activation and an oxidative burst response. AuNS were immediately introduced into the activated human-derived neutrophils incubation system for degradation experiments. The AuNS was synthesized according to the previously reported work and characterized (SI).^14^

### AuNS incubation and long-term degradation assay

AuNS were introduced into the neutrophil culture at a final concentration of 30 µg mL^−1^ on Day 0. Neutrophils were activated using fMLP (100 nM) and Cyt B (5 µg mL^−1^), and freshly added daily for a total duration of 14 days to mimic sustained inflammatory exposure.^16^ Three primary experimental conditions were established: untreated AuNS, Neutrophils + AuNS, and Neutrophils + AuNS in the presence of the myeloperoxidase inhibitor 4-aminobenzoic acid hydrazide (4-ABAH). At Day 14, AuNS samples were centrifuged at 1300 rpm for 1 minute and subjected to structural and elemental characterisation using TEM and SEM-EDX.

### Pharmacological treatment and triple-axis oxidative inhibition

To dissect the individual and combined contributions of distinct neutrophil-derived oxidative pathways to AuNS degradation, additional pharmacological inhibitor conditions were incorporated into the 14 days incubation model. In addition to 4-ABAH (MPO inhibitor, 100 µM), cells were treated with the dual NADPH oxidase 1/4 (NOX1/NOX4) inhibitor GKT137831 (10 µM) and the thioredoxin-1 (Trx-1) inhibitor PX12 (10 µM), administered both individually and in combination. Five inhibitor conditions were examined in parallel: (i) 4-ABAH alone, (ii) GKT137831 alone, (iii) PX12 alone, (iv) 4-ABAH + GKT137831, and (v) 4-ABAH + GKT137831 + PX12 and AuNS alone. All inhibitors were introduced concurrently with neutrophil activation at Day 0 and replenished daily, along with fresh neutrophil addition, throughout the 14 days experimental period. Structural preservation and surface morphological changes were assessed by TEM and SEM, respectively, as described below.^8,17^

Inhibitor combinations followed the sequential organisation of the neutrophil oxidative cascade (NOX-derived ROS to MPO-derived oxidants to Trx-1-dependent thiol capture) rather than a full combinatorial matrix. Conditions were built by stepwise addition along this axis, each inhibitor alone, dual inhibition of the ROS-generating arms, then triple-axis inhibition including the thiol-capture step; since combinations leaving NOX or MPO unblocked would obscure the downstream contributions.

### Scanning electron microscopy coupled with energy-dispersive X-ray spectroscopy (SEM-EDX)

On respective days, the conditioned supernatants containing AuNS and their degradation products were carefully aspirated without disturbing the underlying cells. Supernatants were centrifuged to recover particulate material, and the resulting pellet was gently washed with distilled water to eliminate residual media components, followed by graded ethanol dehydration (30–90%). The dehydrated material was mounted onto aluminium stubs using carbon adhesive and allowed to air-dry completely to avoid artefactual surface distortion. SEM imaging was conducted using a Hitachi S-3400N under high vacuum at an accelerating voltage of 15 kV to resolve nanosheet surface architecture and ultrastructural alterations with high fidelity. For elemental analysis, EDX spectra were acquired from selected regions using Hitachi S3400 corresponding to intact and structurally transformed nanosheet domains.

### Transmission electron microscopy (TEM)

TEM imaging was carried out using a high-resolution transmission electron microscope (ThermoScientific Talos L120C) operated at an accelerating voltage of 120 kV. Multiple regions of each grid were examined to ensure representative imaging of the nanosheet structures. Structural features, including nanosheet integrity, fragmentation, and the presence of diffuse electron-dense regions were analysed at different magnifications. High-magnification images were acquired to resolve nanoscale structural domains and particle assemblies associated with neutrophil-mediated nanosheet transformation.

### HR-TEM analysis

For HR-TEM imaging of AuNS, the samples were drop-cast on a 300-mesh carbon-coated copper grid (Ted Pella, Inc.) and dried for 15–20 min under an infrared (IR) lamp. The samples were imaged using FEI Tecnai G2 F30 S-Twin TEM 300 kV. The chemical composition of the samples was analyzed using energy-dispersive X-ray spectroscopy (EDS) coupled with HR-TEM.

### RNA isolation and transcript expression analysis by quantitative PCR

To assess the early transcriptional inflammatory response of neutrophils to AuNS exposure and inhibitor treatment, cells were collected at 6 hours post-treatment. This time point was selected to capture peak early-response mRNA expression prior to significant neutrophil apoptosis, which occurs progressively under daily activation conditions. Total RNA was isolated from pelleted cells using a validated RNA extraction kit (TRizol, Takara) according to the manufacturer's instructions. RNA concentration and purity were assessed by spectrophotometry (*A*_260_/*A*_280_ ≥ 1.8). Complementary DNA (cDNA) was synthesized from 1 µg of total RNA using a reverse transcription kit under standard cycling conditions. qPCR was performed using SYBR Green. Pro-inflammatory [*TNFα*, *IL6*], and anti-inflammatory [*IL10*, *TGFB1*] genes were included. Expression data were normalized to the geometric mean of validated reference genes [*GAPDH*]. Relative quantification was calculated using the 2^−ΔΔ*C*_t_^ method. All samples were analyzed in technical triplicates from at least three independent biological replicates, and results are expressed as mean ± SD. (Primer sequences, SI Table 1).

### Statistical analysis

Results are presented as mean ± SD of at least three independent biological replicates. Statistical significance was assessed using unpaired Student's *t*-test (for two-group comparisons) and two-way analysis of variance (ANOVA) followed by Tukey's multiple comparisons test (for multi-group comparisons). Significance thresholds were set as follows: *p < 0.05, **p < 0.01, ***p < 0.001, and ****p < 0.0001. All analyses were performed using GraphPad Prism v10.4.1.

## Results and discussion

### Activated neutrophil secreted MPO is indispensable for AuNS degradation

Firstly, the AuNS prepared were characterized by UV-Vis-NIR spectroscopy, HR-TEM, atomic force microscopy (AFM) imaging, dynamic light scattering (DLS) and zeta potential measurements as reported in our previous work.^4^

**Fig. 1:**
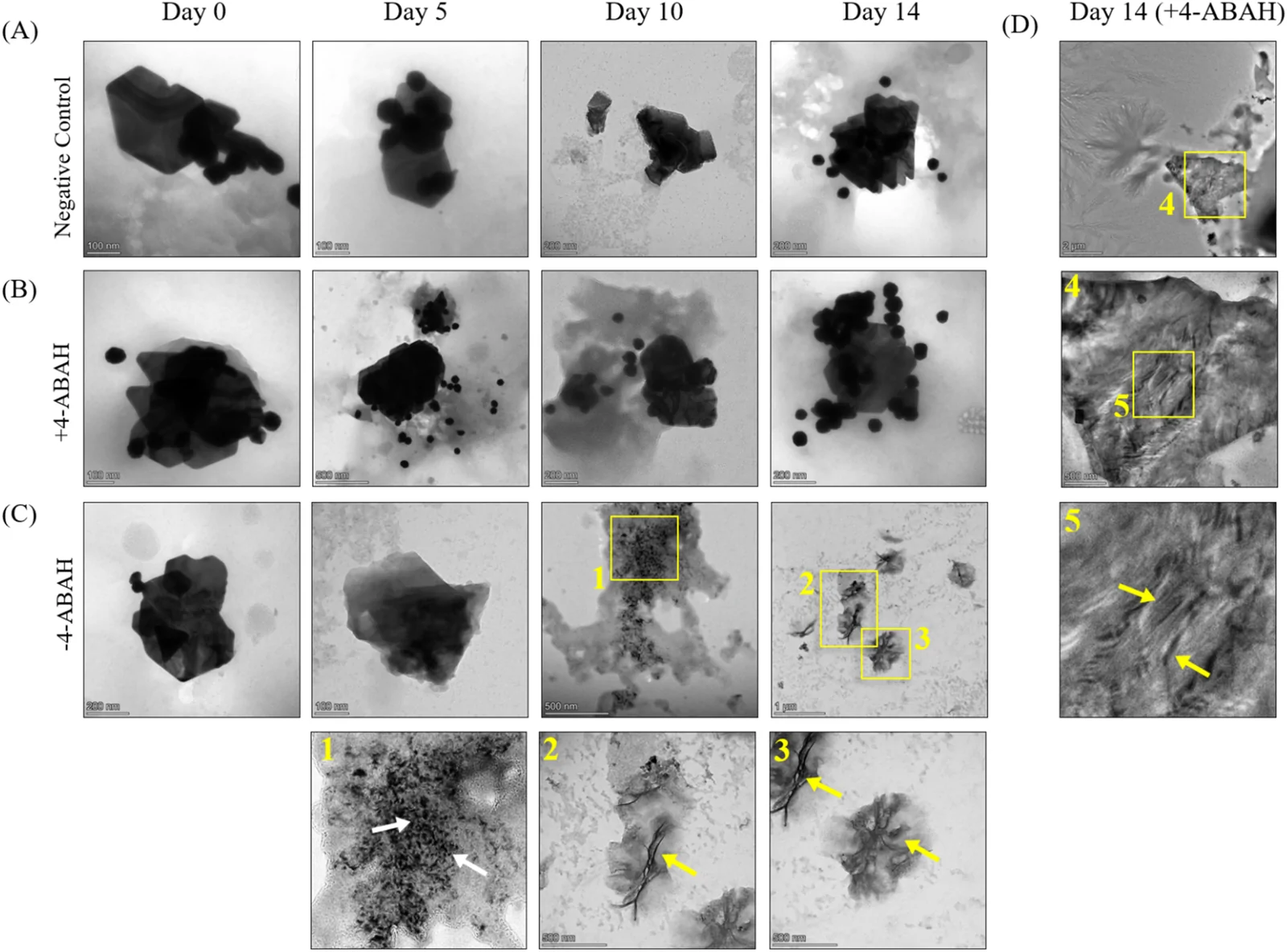
Time-course TEM analysis of AuNS during co-incubation with activated human neutrophils. (A–C) Representative TEM micrographs of AuNS (30 µg mL^−1^) acquired at Day 0, 5, 10 and 14 of incubation. (A) Untreated: AuNS incubated in culture medium alone, showing sheets that retain sharply defined edges and uniform electron density throughout the incubation. (B) AuNS co-incubated with freshly isolated, fMLP/Cyt B-activated neutrophils in the presence of the myeloperoxidase inhibitor 4-ABAH (+4-ABAH, 100 µM). (C) AuNS co-incubated with activated neutrophils in the absence of 4-ABAH (-4-ABAH). Yellow boxes in C (Day 10 and Day 14) mark the fields shown at higher magnification in the numbered panels below: inset 1, Day 10; insets 2 and 3, Day 14. White arrows, electron-dense crystalline domains; yellow arrows, diffuse, low-density regions. (D), AuNS at Day 14 in the presence of 4-ABAH (+4-ABAH), shown at progressively higher magnification. The yellow box (4) in the upper panel is magnified in the middle panel, in which a second yellow box (5) is magnified in the lower panel; yellow arrows indicate the diffuse texture resolved at the highest magnification. Neutrophils were replenished daily for the duration of the incubation. All micrographs were acquired at an accelerating voltage of 120 kV. Scale bars, as indicated in each panel.

To investigate whether neutrophils can induce structural degradation of AuNS, freshly isolated human neutrophils were incubated with AuNS over a 14 days period, replenished every day.^4^ TEM was used to monitor nanosheet morphology at different stages of incubation. At the initial stage, AuNS displayed well-defined sheet-like structures with sharp edges and high electron density, characteristic of intact crystalline gold nanosheets. At Day 0, AuNS displayed well-defined sheet-like structures with sharp edges and uniform electron density, characteristic of intact crystalline gold nanosheets, appearing compact and continuous with no detectable surface irregularities. Upon incubation with neutrophils, progressive structural changes were observed. Several nanosheets exhibited localised regions of reduced electron density and a diffuse morphology, particularly along their edges and surface regions of the nanosheets on Day 10. These diffuse structures appeared as granular or loosely organised domains surrounding partially preserved nanosheet fragments. In contrast, some regions of the nanosheets retained their original dense crystalline appearance, suggesting heterogeneous degradation where intact and transformed domains coexisted within the same structure (Fig. 1A).

At later stages of incubation, the diffuse regions became more prominent and extended further into the nanosheet body, indicating progressive structural destabilisation. High-magnification TEM imaging revealed that these diffuse areas consisted of clusters of smaller electron-dense particles and fragmented nanosheet segments rather than continuous crystalline sheets. Diffuse regions with nanoleaf-like morphology were clearly visible on Day 14 in the primary neutrophil group, whereas no equivalent degradation was observed in the untreated or inhibitor-treated conditions (Fig. 1A). This morphological transformation indicates that neutrophil-derived oxidative mechanisms gradually disrupt the nanosheet lattice, leading to partial fragmentation and structural remodelling of gold nanomaterials.

### MPO inhibition preserves nanosheet morphology but does not abolish degradation

**Fig. 2:**
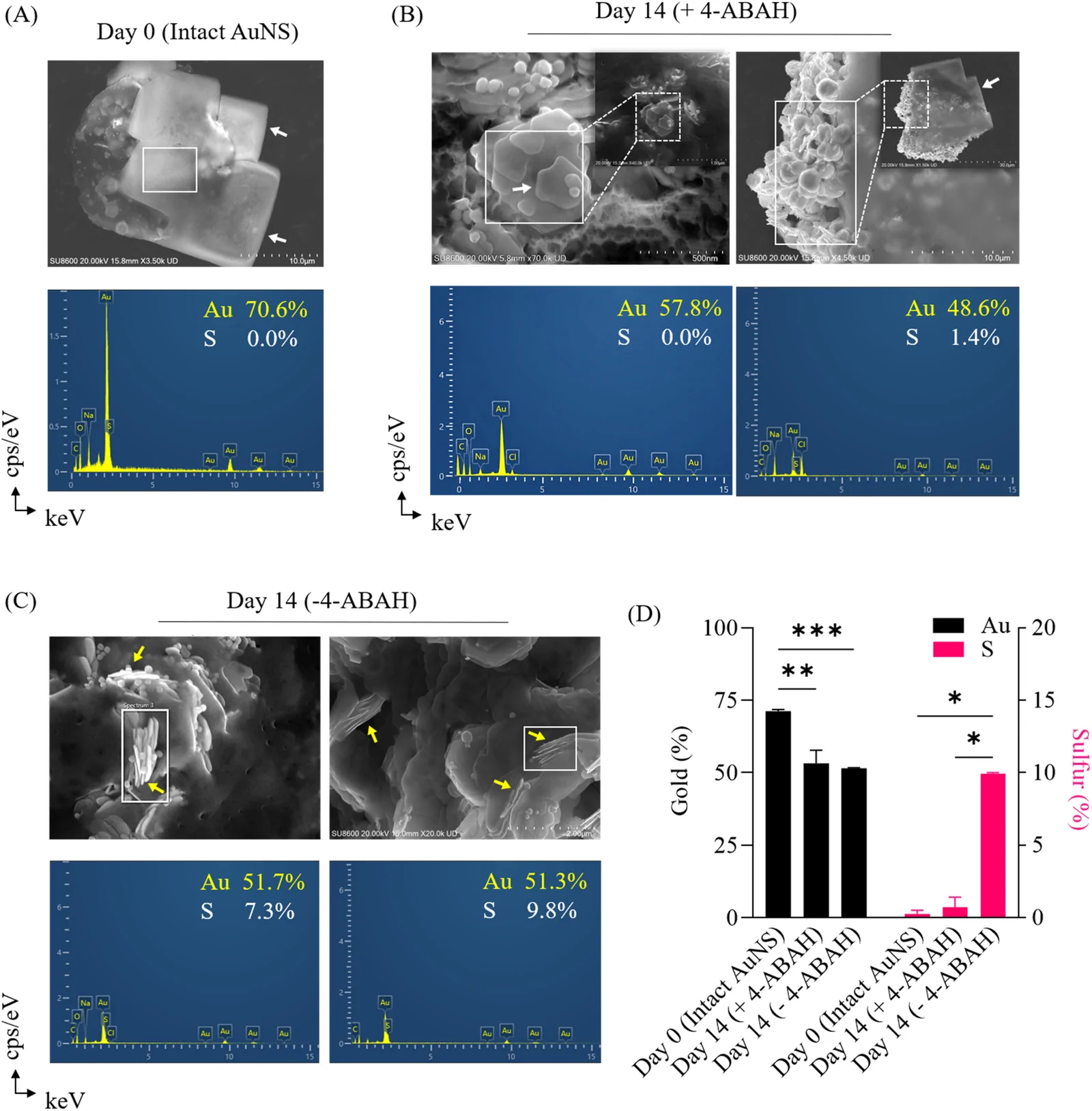
| SEM-EDX profiling of AuNS following co-incubation with human primary neutrophils either in presence or absence of 4-aminobenzoic acid hydrazide (4-ABAH). (A) Representative SEM micrograph of intact AuNS at Day 0 (white arrows, sheet edges) with the corresponding energy-dispersive X-ray (EDX) spectrum acquired from the boxed region; atomic percentages of Au and S are indicated. (B) Micrographs of AuNS after 14 days of co-incubation with neutrophils in the presence of the myeloperoxidase inhibitor (+4-ABAH, 100 µM), shown at two representative fields. Insets show magnified views of the boxed areas (white arrows); EDX spectra were acquired from the boxed regions. (C) Micrographs of AuNS after 14 days of co-incubation with neutrophils in the absence of 4-ABAH (−4-ABAH), shown at two representative fields (yellow arrows). EDX spectra were acquired from the boxed regions. (D) Qualitative analysis of Au (left axis) and S (right axis) atomic percentages for the conditions in A–C. Data are mean ± s.d. (*n* = 3 fields per condition). *P* values were determined by one-way ANOVA with Tukey's multiple-comparison test; *P < 0.05, **P < 0.01, ***P < 0.001. Scale bars, as indicated.

To determine whether MPO contributes to AuNS transformation, incubations were compared by TEM in the presence and absence of 4-ABAH across four time points (Fig. 1). Without inhibitor, sheets at Day 0 and Day 5 remained uniformly electron-dense with sharply resolved edges (Fig. 1c). By Day 10 the sheet no longer presented a defined boundary but a broad, low-density mass containing dispersed small electron-dense particles (Fig. 1C, inset 1, white arrows), and by Day 14 the residual dense material had reorganised into branched, fern-like extensions radiating from a denser core (Fig. 1C, insets 2 and 3, yellow arrows). None of these features developed under MPO inhibition, where sheets retained continuous electron density and unbroken edges throughout (Fig. 1B and D). Consistent with this, sulfur was 0.0% and 1.4% under 4-ABAH against 7.3% and 9.8% in its absence (Fig. 2B–D). Protection was nonetheless incomplete, with gold recovering only to 67.85% against 61.7% untreated and 94.0% intact (Fig. 5C and H), indicating that MPO-independent oxidative activity persists.

### Neutrophil-driven sulfur remodeling of AuNS

To further examine the chemical changes associated with nanosheet degradation, SEM-EDX was performed on samples collected during the incubation period. SEM imaging revealed nanosheet surfaces with diffuse nanoleaf-like structures and particulate aggregates after prolonged incubation with neutrophils (Fig. 2A). Elemental mapping and spectral analysis confirmed the presence of gold as the dominant element in all samples. However, a notable increase in sulfur signals was detected in samples collected after 14 days of incubation in the absence of MPO inhibition. Qualitative EDX analysis demonstrated that sulfur concentrations were significantly elevated in the 14 days samples compared with earlier time points or inhibitor-treated samples (Fig. 2A and B). The sulfur enrichment was particularly evident in regions corresponding to diffuse nanosheet structures, suggesting a mechanistic link between structural degradation and sulfur incorporation. The appearance of sulfur in degraded regions likely reflects interactions between oxidised gold species and sulfur-containing biomolecules present in neutrophils or extracellular proteins. Such sulfur-associated gold assemblies have previously been observed during intracellular gold nanoparticle biotransformation, where oxidative dissolution of gold leads to the formation of biomineralised gold structures enriched in sulfur species.^5,6^ In contrast, samples treated with MPO inhibitor exhibited markedly reduced sulfur signals, further supporting the central role of MPO-mediated oxidative processes in driving nanosheet transformation.

**Fig. 3:**
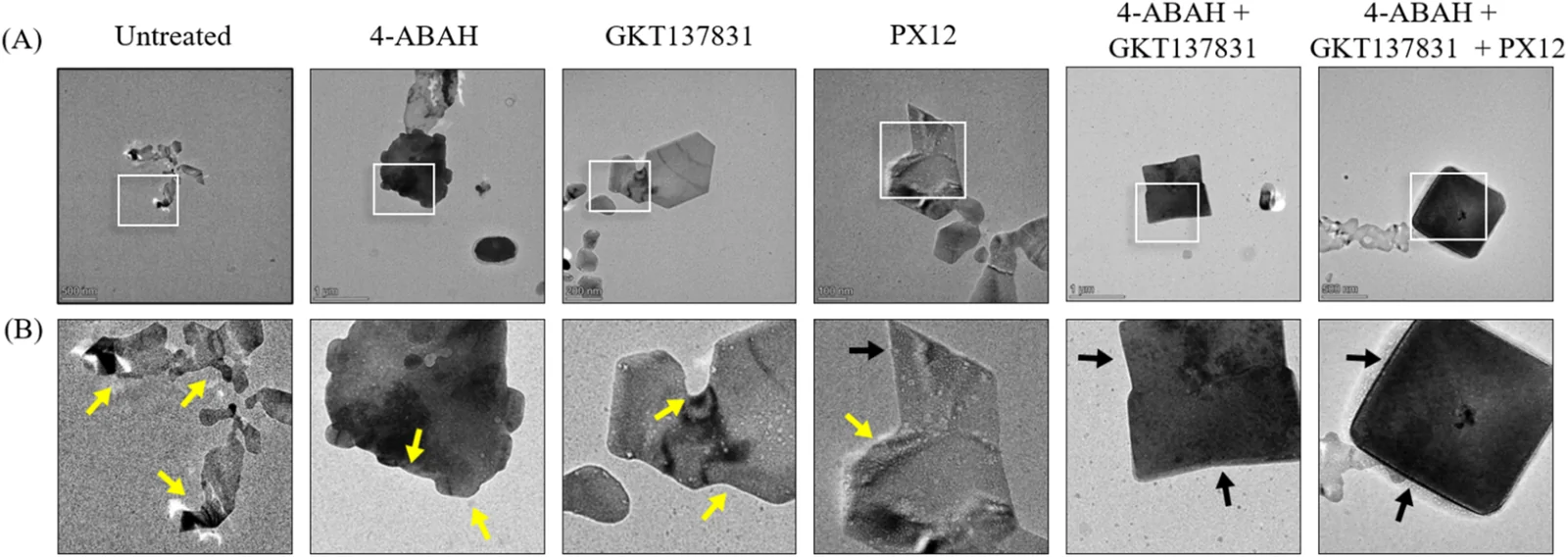
| TEM analysis of AuNS following co-incubation with activated human neutrophils under single and combined triple-axis oxidative-pathway inhibition. (A) Representative TEM micrographs of AuNS collected at Day 14 after co-incubation with neutrophils activated with *N*-formyl-methionyl-leucyl-phenylalanine (fMLP, 100 nM) and cytochalasin B (Cyt B, 5 µg mL^−1^), in the absence of inhibitor (untreated) or in the presence of mechanistically distinct oxidative-pathway inhibitors applied individually or in combination: 4-aminobenzoic acid hydrazide (4-ABAH; myeloperoxidase inhibitor, 100 µM); GKT137831 (setanaxib; dual NADPH oxidase NOX1/NOX4 inhibitor, 10 µM); PX12 (1-methylpropyl 2-imidazolyl disulfide; thioredoxin-1 inhibitor, 10 µM); 4-ABAH + GKT137831; and 4-ABAH + GKT137831 + PX12. Inhibitors were added concurrently with neutrophil activation at Day 0 with fresh neutrophils for the duration of the incubation. White boxes indicate the fields shown at higher magnification in (B). Yellow arrows: regions of structural degradation; black arrows: structurally intact regions. Micrographs were acquired at an accelerating voltage of 120 kV. Scale bars, as indicated.

Given that MPO activity is fueled by upstream ROS generation and that residual AuNS degradation persisted despite MPO inhibition, we next investigated whether additional neutrophil oxidative pathways contribute to AuNS degradation, a systematic pharmacological dissection was performed using three mechanistically distinct inhibitors: 4-ABAH (MPO), GKT137831 (NADPH oxidase NOX1/NOX4), and PX12 (thioredoxin-1). TEM analysis at Day 14 revealed a hierarchical pattern of structural protection across the five inhibitor conditions (Fig. 3A and B). Individual inhibitor treatment with either GKT137831 or PX12 resulted in partial preservation of nanosheet morphology, with fewer and less extensive diffuse regions compared with uninhibited neutrophil-AuNS incubations. Consistent with earlier observations, 4-ABAH alone conferred substantial protection. The dual combination of 4-ABAH and GKT137831 further reduced degradation, with the majority of nanosheets retaining intact crystalline domains and sharp structural boundaries, indicating an additive or synergistic interaction between MPO and NOX-derived ROS in mediating AuNS structural disruption.

**Fig. 4:**
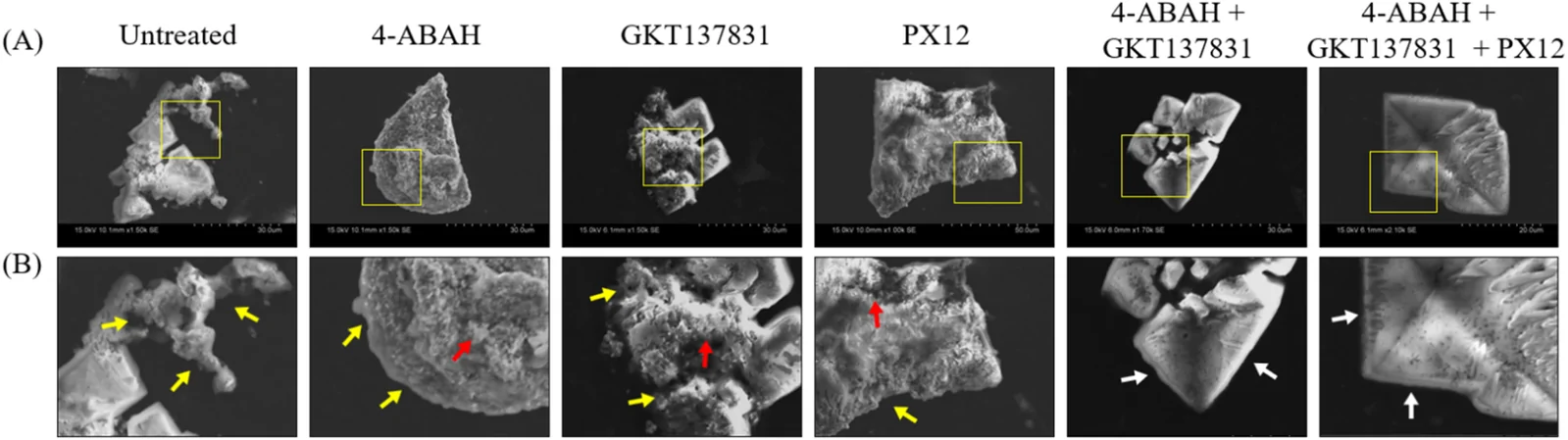
Triple-axis oxidative-pathway inhibition and sulfur chelation suppresses neutrophil mediated degradation of gold nanostructures. (A) Representative SEM micrographs of AuNS collected at Day 14 after co-incubation with activated human neutrophils in the absence of inhibitor (untreated) or in the presence of 4-ABAH, GKT137831, PX12, 4-ABAH + GKT137831, or 4-ABAH + GKT137831 + PX12. Yellow boxes indicate the fields shown at higher magnification in (B). Yellow arrows, regions of structural degradation; red arrows, surface abrasion; white arrows, structurally intact regions. Micrographs were acquired at an accelerating voltage of 15 kV. Scale bars, as indicated.

The greatest degree of structural preservation was observed under triple-axis inhibition with 4-ABAH + GKT137831 + PX12 (Fig. 3A and B). Under these conditions, nanosheets closely resembled untreated controls, displaying well-defined edges, continuous electron-dense crystalline domains, and a near-complete absence of diffuse degradation regions. These findings demonstrate that the full spectrum of neutrophil-mediated AuNS degradation requires the convergent activity of MPO-derived oxidants, NOX-generated superoxide and hydrogen peroxide, and thioredoxin-dependent redox processes.^8,17,18^ The contribution of PX12 to the protective effect of triple-axis inhibition suggests that thioredoxin-1 activity and its downstream redox targets participate in the oxidative remodelling of gold nanostructures, potentially by sustaining the regeneration of reduced glutathione or other thiol-dependent species that facilitate gold oxidation. Importantly, SEM-EDX elemental analysis of all inhibitor conditions at Day 14 revealed a hierarchical reduction in sulfur incorporation that closely mirrored the pattern of structural protection observed by TEM (Fig. 4). Uninhibited neutrophil-AuNS samples at Day 14 showed prominent sulfur peaks in EDX spectra localised to structurally disrupted nanosheet domains. Treatment with PX12 alone produced a moderate but detectable reduction in sulfur signal intensity relative to uninhibited neutrophil-AuNS samples, indicating that thioredoxin-1-dependent redox activity directly contributes to sulfur incorporation, likely by maintaining pools of reduced thiol species that serve as binding partners for oxidatively dissolved gold intermediates. GKT137831 treatment produced a comparable intermediate reduction in sulfur signals, consistent with the partial morphological protection observed by TEM, confirming that NADPH oxidase-derived ROS also drive sulfur-gold biotransformation. The dual combination of 4-ABAH and GKT137831 produced a pronounced suppression of sulfur signals, whereas the complete triple-axis inhibition with 4-ABAH + GKT137831 + PX12 produced a near-complete abolition of detectable sulfur peaks in EDX spectra. These data establish that sulfur incorporation into degraded nanosheet domains is a direct and sensitive readout of multi-pathway oxidative activity, requiring the convergent engagement of MPO, NADPH oxidase, and thioredoxin-dependent pathways, and is not attributable to passive adsorption of sulfur-containing proteins onto intact nanosheet surfaces.

Complementary SEM analysis of samples from all five inhibitor conditions confirmed the TEM findings and provided additional surface morphological detail (Fig. 4). Uninhibited neutrophil-AuNS samples at Day 14 displayed pronounced surface roughening, edge erosion, and structurally heterogeneous domains with extensive nanoleaf-like protrusions. Single-inhibitor conditions showed intermediate surface preservation, whereas the 4-ABAH + GKT137831 combination substantially reduced surface disruption. Triple-axis inhibition with 4-ABAH + GKT137831 + PX12 produced surfaces most closely resembling intact untreated nanosheets, with smooth morphology, well-defined edges, and minimal structural heterogeneity. Taken together, the TEM and SEM data establish that neutrophil-mediated AuNS degradation is driven by the coordinated activity of multiple oxidative pathways, and that comprehensive structural protection requires simultaneous suppression of all three axes.

### HR-TEM and EDS analysis reveals sulfur accumulation correlates with inhibitor-dependent gold preservation

**Fig. 5:**
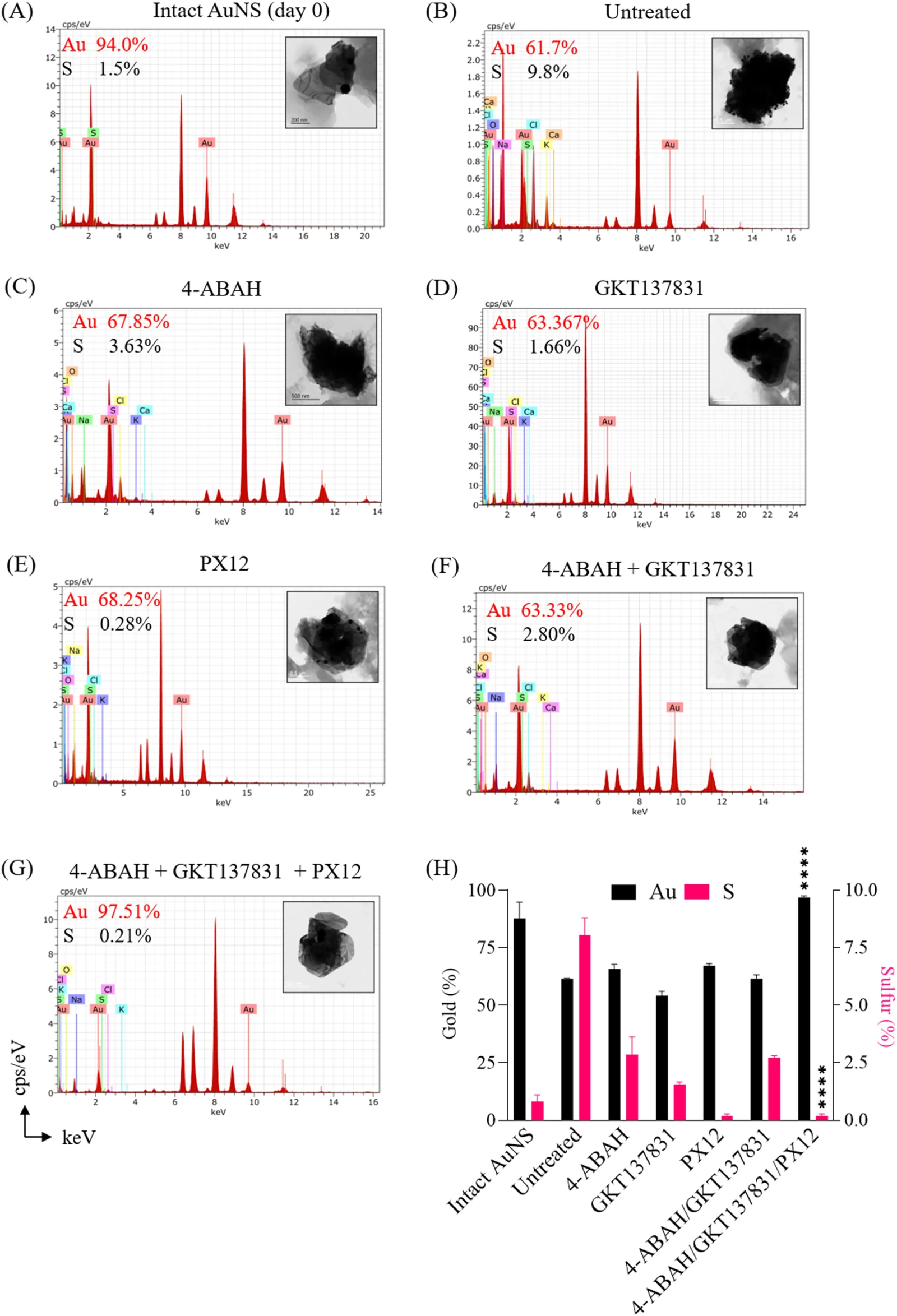
HR-TEM and EDX analysis of AuNS across single and combined oxidative-pathway inhibition at Day 14. (A–G) Representative energy-dispersive X-ray (EDX) spectra acquired from AuNS under each condition, with the corresponding HR-TEM micrograph of the analysed particle shown as an inset. Conditions are: intact AuNS at Day 0 (A); AuNS co-incubated with activated human neutrophils in the absence of inhibitor (untreated, B); and AuNS co-incubated with activated neutrophils in the presence of 4-ABAH (C), GKT137831 (D), PX12 (E), 4-ABAH + GKT137831 (F), or 4-ABAH + GKT137831 + PX12 (G). Atomic percentages of Au and S obtained from each spectrum are indicated on the corresponding panel; labelled peaks denote the elements assigned during spectral deconvolution. Scale bars, as indicated in the insets. (H) Qualitative of Au (left axis) and S (right axis) atomic percentages across all conditions. Data are mean ± s.d. (*n* = 3 independent measurements per condition). *P* values were determined by two-way ANOVA with Tukey's multiple-comparison test, comparing each group with the untreated conditions for both elements; ****P < 0.0001. Micrographs and spectra were acquired at an accelerating voltage of 300 kV.

To directly qualitatively analyse the elemental changes underlying inhibitor-dependent gold preservation, HR-TEM coupled with EDS was performed on AuNS from all conditions at Day 14 (Fig. 5). Intact AuNS (Day 0) exhibited an Au atomic percentage of 94.0% and a baseline sulfur content of 1.5% (Fig. 5A). Following 14 days of untreated PMN exposure, Au content declined to 61.7% accompanied by a 6.53-fold rise in sulfur to 9.8% (Fig. 5B), consistent with oxidative dissolution of Au^0^ and subsequent complexation of soluble gold intermediates with biological thiol species. Single-agent inhibition revealed that gold preservation and sulfur incorporation are pharmacologically dissociable rather than co-varying readouts. All three inhibitors conferred only marginal recovery of gold content, restoring Au to 67.85% (4-ABAH), 63.37% (GKT137831) and 68.25% (PX12), an improvement of no more than 6.6 percentage points over the untreated condition and far short of the intact baseline (Fig. 5C–E). Sulfur suppression, by contrast, was strongly pathway-dependent and followed a distinct rank order: 4-ABAH reduced sulfur 2.70-fold to 3.63%, GKT137831 5.90-fold to 1.66%, and PX12 35-fold to 0.28%, essentially to background. That PX12 alone abolishes the Au-S signature whilst leaving bulk gold loss largely unaltered identifies thioredoxin-1 as the downstream thiol-capture step rather than an oxidative one, its active-site cysteines providing the acceptor that retains oxidatively generated Au^+^ as surface-associated Au(i)-thiolate. Conversely, the persistence of readily detectable sulfur under 4-ABAH confirms that MPO-independent thiol-oxidising activity remains operative when the granule-derived arm alone is suppressed. Dual blockade of MPO and NADPH oxidase (4-ABAH + GKT137831) suppressed sulfur 3.50-fold to 2.80% yet left Au at 63.33% (Fig. 5F), indicating that simultaneous inhibition of both ROS-generating arms is insufficient for gold preservation whilst the downstream capture step remains available. Critically, addition of PX12 to achieve triple-axis inhibition drove Au content to 97.51% and reduced sulfur to 0.21% (Fig. 5G), a further 10-fold suppression beyond the dual-inhibitor condition and comparable to the intact baseline of 1.5%; the marginal excess of Au relative to Day 0 material falls within the variation expected of localised spot EDS analysis and should not be interpreted as enrichment. The near-complete abolition of sulfur incorporation under triple-axis blockade, alongside near-intact Au preservation, establishes that convergent engagement of MPO, NADPH oxidase, and thioredoxin-1 is cooperatively required to drive AuNS biotransformation, and that simultaneous suppression of all three axes is required to achieve complete structural and elemental preservation (Fig. 5A–H).

### Differential regulation of inflammatory mediators under inhibitor treatment conditions

**Fig. 6:**
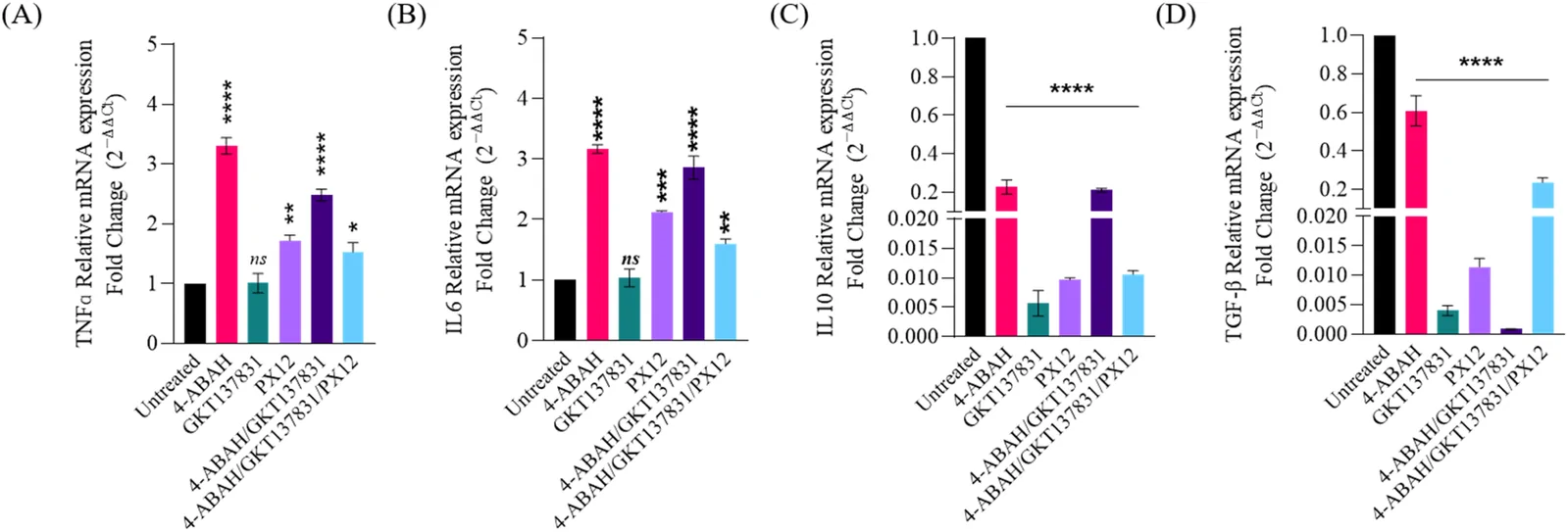
Differential regulation of pro- and anti-inflammatory mediators under experimental treatment conditions. (A and B) Quantitative expression analysis of pro-inflammatory cytokines following treatment, illustrating the activation or suppression of inflammatory signaling pathways across experimental groups. (C and D) Expression profiles of anti-inflammatory mediators' response elicited under the respective treatment conditions. All data are expressed as mean ± SD, independent biological replicates. Statistical significance was assessed using two-way analysis of variance (ANOVA) followed by Tukey's multiple comparisons test. Significance thresholds were set as follows: *p < 0.05, **p < 0.01, ***p < 0.001 and ****p < 0.0001. All analyses were performed using GraphPad Prism v10.4.1.

To evaluate whether the neutrophil inflammatory programme is coupled to the oxidative pathways mediating AuNS structural degradation, mRNA expression of pro- and anti-inflammatory cytokines was quantified in freshly isolated neutrophils from all experimental conditions at 6 hours post-treatment (Fig. 6). Neutrophils in the untreated neutrophil + AuNS condition exhibited significantly elevated expression of pro-inflammatory cytokines TNF-α and IL-6 (Fig. 6A and B), consistent with active neutrophil activity and oxidative activation in response to nanosheet exposure. This robust pro-inflammatory transcriptional response in human primary neutrophils provides direct immunological evidence that the neutrophils are recognising and actively processing the AuNS rather than remaining passive bystanders.

TNF-α and IL-6 levels remained elevated relative to untreated controls across all inhibitor-treated conditions, indicating persistent inflammatory activation. However, cytokine expression exhibited a progressive decline with increasing inhibitor complexity. While treatment with 4-ABAH alone and the 4-ABAH + GKT137831 combination was associated with higher TNF-α and IL-6 levels, the addition of PX12 to generate triple-axis inhibition (4-ABAH + GKT137831 + PX12) resulted in a marked reduction in both cytokines relative to the other inhibitor groups, consistent with the enhanced structural preservation of AuNS observed by TEM.

Anti-inflammatory cytokine expression revealed a congruent rather than reciprocal pattern: both IL-10 and TGF-β1 were also significantly reduced under inhibitor treatment conditions (Fig. 6C and D), indicating that suppression of the oxidative burst broadly attenuates the overall neutrophil inflammatory programme rather than selectively dampening pro-inflammatory signaling alone. The coordinated reduction of both cytokine classes under inhibitor treatment demonstrates that the pro- and anti-inflammatory responses of primary human neutrophils are co-regulated by the same oxidative axes, MPO, NADPH oxidase, and thioredoxin-1 that mediate AuNS structural degradation. Triple-axis inhibition produced the most pronounced suppression, with IL-10 and TGF-β1 expression reaching near-basal levels consistent with the near-complete abolition of oxidative burst activity and nanosheet degradation observed under 4-ABAH + GKT137831 + PX12 treatment. Taken together, the parallel attenuation of both structural nanosheet degradation (TEM and SEM-EDX) and cytokine-mediated inflammatory responses by identical pharmacological inhibitors in freshly isolated human primary neutrophils establishes a mechanistic coupling between neutrophil oxidative burst activity and AuNS biotransformation, providing dual structural and immunological evidence that human primary neutrophils are active mediators of gold nanosheet degradation.

While each single inhibitor afforded only partial protection (Au content 63–68% *vs.* 61.7% in untreated, *vs.* 94% intact), PX12 selectively collapsed the surface sulfur signal to background (9.8% → 0.25%), confirming Trx1 as the source of the Au–S-TrX thiol adducts. Net dissolution was abolished only by triple-axis blockade: combining PX12 with dual MPO/NOX inhibition (4-ABAH + GKT137831) restored Au content to 97.5% with sulfur at 0.25%, yielding near-complete nanosheet persistence (Fig. 5B).

The present work directly extends our previously published investigation of gold nanosheet biotransformation,^4^ in which we first demonstrated that two-dimensional (2D) AuNS undergo progressive structural remodelling upon cellular interaction, characterised by the formation of diffuse electron-dense degradation products associated with sulfur-containing biomolecules. The study established the fundamental capacity of cells to mediate oxidative transformation of gold nanomaterials and identified sulfur incorporation as a hallmark of AuNS biotransformation in the cellular environment. The current investigation advances this paradigm by introducing human PMN neutrophils as a uniquely potent biological model, exploiting their multi-enzyme oxidative mechanism encompassing MPO, NADPH oxidase, and thioredoxin-dependent pathways to dissect the relative contributions of individual and combined oxidative axes to nanosheet structural transformation. Unlike the cell types examined in our earlier work,^4^ primary neutrophils deploy convergent, pharmacologically dissociable oxidative mechanisms that each contribute independently and synergistically to nanosheet structural transformation, necessitating simultaneous inhibition of all three axes for complete nanosheet protection. The sulfur enrichment in degraded nanosheet domains detected by SEM-EDX in the present study is mechanistically consistent with the previous findings,^5^ demonstrating that oxidative dissolution of gold nanoparticles generates soluble gold intermediates that form thermodynamically stable complexes with biological thiol groups and sulfur-containing proteins, ultimately yielding biomineralised gold-sulfur assemblies. The observation in the present study that triple-axis pharmacological inhibition abolishes sulfur incorporation alongside structural preservation provides direct evidence that neutrophil-derived oxidative activity is the primary driver of this sulfur–gold interaction, rather than passive adsorption of sulfur-containing proteins onto nanosheet surfaces, thus extending the sulfur-mediated biotransformation mechanism originally characterised in the previous study^5^ to 2D gold nanosheet geometry under immune cell activation conditions.

Neutrophils were selected as the initial biological interface for systemically administered nanomaterials, whereas tissue-resident macrophages engage them only downstream. MPO is markedly downregulated upon monocyte-to-macrophage differentiation.^19,20^ Although MPO-positive populations can persist under specific cytokine environments, macrophages, while retaining NOX2-derived ROS and an intact thioredoxin system, would be expected to support the Fenton-like and thiol-capture steps but not the HOCl-driven lattice disruption reported here. Direct comparison of the two cell types is an important extension of this work.

## Conclusion

This study demonstrates that human neutrophils mediate progressive structural degradation of AuNS through a multi-pathway oxidative mechanism. Sustained 14 days exposure of AuNS to freshly isolated, activated neutrophils without any inhibitory treatment resulted in pronounced structural remodelling, characterised by the loss of nanosheet crystallinity, formation of diffuse electron-dense domains, and a significant increase in sulfur incorporation as confirmed by SEM-EDX elemental analysis. The emergence of sulfur-enriched degradation products is consistent with oxidative dissolution of Au^0^ into soluble gold intermediates that subsequently form stable complexes with biological thiol groups and sulfur-containing biomolecules in the neutrophil microenvironment.^4,5^ These findings confirm that the neutrophil oxidative microenvironment is sufficient to drive biotransformation of structurally intact gold nanomaterials under physiologically relevant inflammatory conditions.

Selective inhibition of MPO with 4-ABAH provided substantial but incomplete protection, indicating that MPO-derived oxidants are important contributors to AuNS transformation while additional oxidative pathways remain active.^8^ The persistence of sulfur incorporation following MPO inhibition further supports contributions from NADPH oxidase-derived ROS and thioredoxin-1-dependent redox processes. Complete structural preservation was observed only following simultaneous inhibition of MPO, NADPH oxidase and thioredoxin-1, accompanied by near-complete suppression of sulfur incorporation. Together, these findings indicate that AuNS biotransformation is not governed by a single oxidative pathway but instead depends on the coordinated activity of multiple neutrophil redox systems.

**Scheme 1:**
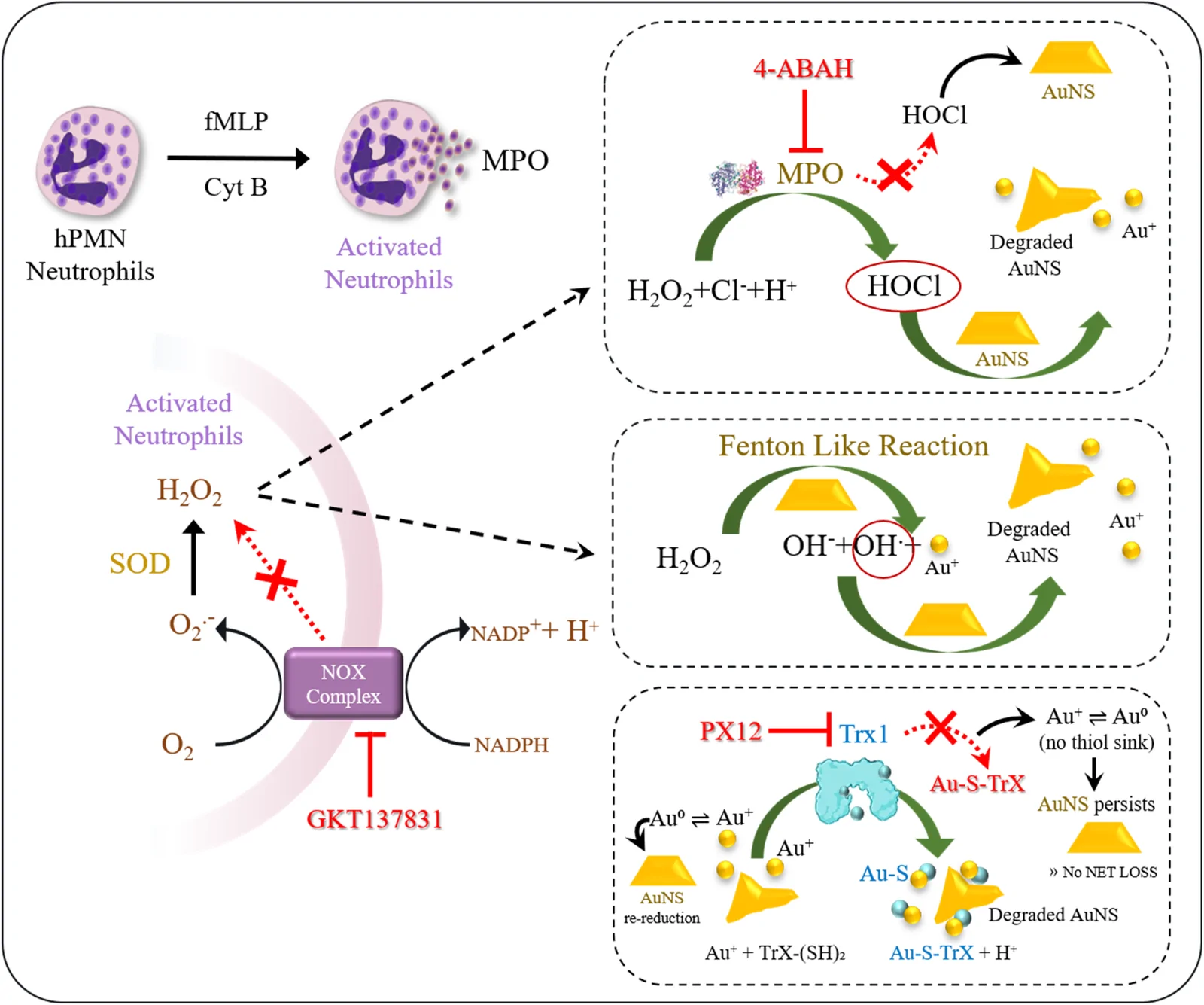
Mechanism of neutrophil-mediated biodegradation of AuNS.

Mechanistically, NADPH oxidase-derived superoxide can generate H_2_O_2_ through dismutation, providing substrate for MPO-dependent production of highly reactive oxidants, including HOCl and associated radical intermediates.^21^ H_2_O_2_ may additionally participate in Fenton-like reactions, generating hydroxyl radicals capable of contributing to AuNS degradation, consistent with our previous observations in gold nanomaterials.^4,5^ Oxidative dissolution of AuNS generates Au^+^ species that can subsequently interact with sulfur-containing redox proteins such as thioredoxin-1 through their active-site cysteine thiols. The marked suppression of sulfur incorporation following PX12 treatment supports a role for thiol-dependent gold capture, consistent with the high affinity of thioredoxin cysteine residues for soft metal ions such as gold.^22^ These complementary processes provide a mechanistic basis for the neutrophil-mediated AuNS transformation proposed in Scheme 1.

The two untested combinations (GKT137831 + PX12 and 4-ABAH + PX12) each leave a major oxidant-producing enzyme active, so degradation would continue through that route and mask the contribution of the blocked pathways. Without these conditions, we cannot rule out compensation between the NOX and thioredoxin-1 pathways when MPO is active. Thus, our results show that the three pathways act together rather than independently.

Taken together, these findings challenge the prevailing assumption that gold nanomaterials are bioinert and identify innate immune cells as important mediators of gold nanostructure degradation in biological environments. Building directly upon our prior work,^4^ which established AuNS biotransformation in non-immune cellular models, the present study reveals that the neutrophil oxidative environment introduces a qualitatively distinct and mechanistically more complex degradation programme. Understanding these immune-mediated degradation pathways is essential for accurately predicting the long-term stability, biodistribution, and immunological consequences of gold-based nanomaterials in biomedical applications, and underscores the importance of incorporating physiologically relevant immune cell models in the preclinical evaluation of nanomaterial safety and performance.

## Ethical statement

Human peripheral blood was collected from healthy volunteer donors with their informed consent, in accordance with the relevant guidelines and regulations. The study protocol was reviewed and approved by the Institutional Ethics Committee of CSIR-Indian Institute of Chemical Technology (IEC approval no. IEC-121/2025).

## Author contributions

S. P., V. N., and D. S. contributed equally to this work. S. P.: methodology, formal analysis, data curation, visualization, writing. V. N.: methodology, formal analysis, writing, data curation, visualization. D. S.: methodology, writing. P. K.: methodology, writing, visualization. R. K.: supervision, review & editing. S. R. B.: conceptualization, supervision, project administration, resources, funding acquisition, review & editing. All authors reviewed and approved the final manuscript.

## Conflicts of interest

There are no conflicts to declare.

## Supplementary Material

NA-OLF-D6NA00592F-s001

## Data Availability

The data that support the findings of this study are available from the corresponding author upon reasonable request. Supplementary information (SI) is available. See DOI: https://doi.org/10.1039/d6na00592f.
