# Supplementary material for: Immune-driven biotransformation of gold nanosheets by human primary neutrophils
Source: Nanoscale Adv. 2026 Aug 28. Online ahead of print. doi: 10.1039/d6na00592f (PMC13523013; doi:10.1039/d6na00592f)
Supplement: Supplementary file 1 [file NA-OLF-D6NA00592F-s001.pdf]

Supplementary Information (SI) for Nanoscale Advances.
This journal is © The Royal Society of Chemistry 2026

Immune-Driven Biotransformation of Gold Nanosheets by Human Primary Neutrophils

Soumyadeep Poddar,[a,b#] Vishal Nayak,[a#] Deeksha Suvarna,[a#] Pavithra Kurungottu,[c] Sandeep Shrivastava,[d] Rajendra Kurapati,[c*] Srinivasa Reddy Bonam[a*]

[a] Vaccine Immunology Laboratory, Department of Applied Biology, CSIR-Indian Institute of Chemical Technology, Hyderabad 500007, India

[b] Academy of Scientific and Innovative Research, Ghaziabad 201002, India

[c] School of Chemistry, Indian Institute of Science Education and Research Thiruvananthapuram, Maruthamala P.O., Vithura, Thiruvananthapuram, Kerala 695551

[d] CSIR-Centre for Cellular and Molecular Biology, Uppal Road, Hyderabad 500 007, India.

* E-mail: bonamsr.iict@csir.res.in; rkurapati@iisertvm.ac.in

[#] equal contribution

Synthesis of tryptophan-reduced gold nanosheets (AuNS)

The AuNS were synthesized as per the reported procedure and characterized in our previous work.[1, 2] The glassware required for the procedure was thoroughly cleaned with aqua regia and dried before the experiment. Following this, 10 mL of 10 mM $HAuCl_4 \cdot 3H_2O$ (Sigma-Aldrich) and 10 mL of 5 mM tryptophan (Tokyo Chemicals; TCI) were mixed in a glass vial and stirred continuously for 1 h at room temperature. The suspension was left at room temperature for 72 h without any disturbance and then freeze-dried. The synthesized AuNS was characterized using UV-vis-NIR spectroscopy, HR-TEM, AFM, DLS and zeta potential measurements.[2]

Table 1. qPCR primer sequences.

Primer	Forward sequence	Reverse sequence
TNFα Human qPCR Primer Pair (NM_000594)	CTCTTCTGCCTGCTGCACTTTG	ATGGGCTACAGGCTTGTCACTC
IL-6 Human qPCR Primer Pair (NM_000600)	AGACAGCCACTCACCTCTTCAG	TTCTGCCAGTGCCTCTTTGCTG
IL-10 Human qPCR Primer Pair (NM_000572)	TCTCCGAGATGCCTTCAGCAGA	TCAGACAAGGCTTGGCAACCCA
TGFβ Human qPCR Primer Pair (NM_000660)	TACCTGAACCCGTGTTGCTCTC	GTTGCTGAGGTATCGCCAGGAA
GAPDH Human qPCR Primer Pair (NM_002046)	GTCTCCTCTGACTTCAACAGCG	ACCACCCTGTTGCTGTAGCCAA

References

1. Baral, A.; Cavalieri, F.; Chattopadhyay, S.; Ashokkumar, M., Synthesis of Gold Nanosheets with Controlled Morphology by Combining a Natural Amino Acid with High-Frequency Ultrasound. *ACS Sustainable Chemistry & Engineering* **2021,** *9* (41), 13953-13962.
2. Kurungottu, P.; Anilkumar, A. K.; Poddar, S.; Swetha, K.; Anil, P.; Bonam, S. R.; Kurapati, R., Biodegradable Aurum: Gold Nanosheets Undergo Biodegradation by Neutrophil Myeloperoxidase. **2026,** *6* (4), e202500491.
